# The Need for Biosecurity Education in Biotechnology Curricula

**DOI:** 10.34133/bdr.0008

**Published:** 2023-03-14

**Authors:** Ying-Chiang J. Lee, Xuanqi Chen, Siddharth Marwaha

**Affiliations:** ^1^ Department of Molecular Biology, Princeton University, Princeton, NJ 08544, USA.; ^2^ Department of Chemical and Biological Engineering, Princeton University, Princeton, NJ, 08544, USA.

## Abstract

The growth of biotechnology in recent decades and the dual-use nature of most bioscience research are making their misuse, or accidental misuse or release, more likely and present as threats to biosecurity. A proactive approach is through educating the next generation of scientists to be more security conscious. However, current educational and professional programs in biosecurity are lacking. In this perspective, we recommend that biosecurity educational opportunities should be implemented and expanded for undergraduate and graduate students who will likely use one or more methods in the field of biotechnology. We then propose that biosecurity education is a key factor in a path toward sustainable and safe research. Finally, a set of 17 biosecurity competencies organized into 6 distinct themes is outlined.

## Introduction

Biotechnology has grown quickly in the past several decades, and advances in gene editing and machine learning approaches have lowered the barrier to the design of harmful biological and chemical agents [1,2]. Growth of open science in the form of open code and preprints has made these methods increasingly accessible to the public [3]. These factors create an environment where researchers could, intentionally or accidentally, create and release dangerous and novel pathogens or toxins and scientists. While this proliferation of biological knowledge has not yet manifested in a large-scale bioterrorism event, scientific progress is steadily decreasing the barrier for bad actors to misuse biotechnology and scientific research in general. Even identifying studies with high dual-use risk is now easier than ever before [4]. This trend has not gone unnoticed by the larger research community, evidenced in part by focused biosecurity publications by the National Academies in the United States [5,6]. Other countries, such as China, are also engaging in similar discourse [7,8]. Although aspects of biosafety, biosecurity, and responsible conduct are common in the literature, the lack of integration leaves room for improvement [9]. A challenge is that enactment of biosecurity policies and legislation may be perceived as reactionary rather than proactive, particularly in light of the COVID-19 pandemic.

One tool can be used that preempts any high-level policy change—education. Educating the next generation of scientists, particularly those in the biotechnology and adjacent fields such as medicine and chemistry, to be responsible is relatively simple to implement among life science and academic settings [10]. Here, education can be a valuable tool in providing researchers with a basic understanding of biosecurity and the risks involved. While nearly every aspect of life science and biotechnology research can be dual use and subject to misuse, educating students on past transgressions in research, what kinds of scientific questions might carry increased security risks, and best practices can help create a first line of defense even before policies are drafted at the institutional, federal, and international levels. Similar approaches in fields such as nuclear safety and security have led to great successes in risk mitigation that could be replicated for biosecurity and biosafety [9]. Here, we highlight the need and approaches to incorporate biosecurity education into bioscience curricula that promotes the safe, sustainable continuation of biotechnology research.

## Education and Professional Programs in Biosecurity are Lacking

The number of institutions offering biosecurity-focused, or biosecurity-adjacent, degrees and concentrations and the number of biosecurity-related career development programs such as fellowships are lacking. Most students that use the tools of biotechnology in laboratory research will not be enrolled in such specialized programs and may never have an opportunity to take a course on biosecurity during their undergraduate or graduate years despite the demonstrated feasibility of implementing biosecurity courses and workshops [10,11]. Professional programs in biosecurity are similarly lacking. Two fellowships, the Emerging Leaders in Biosecurity Fellowship at the Johns Hopkins Center for Health Security and the Fellowship for Ending Bioweapons hosted by the Council on Strategic Risks, offer the opportunity to explore biosecurity risks for those in established careers but draw from a talent pool that has demonstrated interest in biosecurity. Other programs such as the International Genetically Engineered Machine (iGEM) competition incorporate biosecurity themes into the competition itself [12]. The iGEM audience not only spans a growing range of individuals from the high school level to working professionals but is also restricted in reach because attendees must be part of existing iGEM groups at institutions. Additionally, even among iGEM participants, a dual-use education gap was apparent [13]. Although these opportunities are invaluable experiences for students hoping to gain a deeper understand and perhaps become leaders in the field of biosecurity, many undergraduate and graduate students involved in bioscience and biotechnology research are distally situated and may never be adequately introduced to biosecurity topics at a level that will make a lasting impact.

The reach and accessibility of biosecurity education needs to be further developed and broadened. While the COVID-19 pandemic has reignited public interest in biosecurity and pandemic preparedness, general interest in such topics is expected to decline over time, pending a major outbreak caused by yet another pathogen. This waxing and waning of interest combined with the lack of classroom instruction on biosecurity topics limits not only the spread of biosecurity knowledge but also those who can become subject matter experts. To mitigate these issues and facilitate the development of the next generation of security-conscious scientists, biosecurity should be integrated into coursework for undergraduate and graduate students spanning the chemical to biological sciences who will likely use one or more methods found in the field of biotechnology.

## Biosecurity Education for Sustainable Research

Students are underexposed to topics in biosecurity, even when taking courses covering research ethics and responsibility. Graduate programs in the United States funded by the National Institutes of Health (NIH) are required to provide responsible conduct in research training that covers subjects such as data fabrication, conflicts of interest, and ethics [14]. Students may occasionally encounter case studies in, or tangential to, synthetic biology and genetic engineering. However, the responsible application and pursuit of research questions in terms of biosecurity are not included in these required topics. The implications for lapses in both biosecurity and bioethics for the field of biotechnology and science in general are also often neglected. Furthermore, academics and industry professionals alike often encounter research ethics justifications when applying for funding through government programs like the NIH or the National Science Foundation (NSF), and through this pathway, even professionals who were never exposed to the mandated training are made to learn about responsible research practice [15,16].

Integration of educational materials into the NIH’s existing coursework structure provides a natural way to expose graduate students to biosecurity concepts. Likewise, integration of biosafety sections into NIH, NSF, and other grants provides this information to researchers later into their career and whom may have missed this education at earlier stages. Continued reinforcement of core biosafety concepts throughout one’s career will further the overall understanding and implementation of these concepts. Furthermore, the increased need for this education will lead to the development of quality educational resources, which will, in turn, further improve the understanding of these topics.

Ultimately, guiderails to ensure the sustainability of research as an institution are more important than any given project, field, or technique. Over time, sustainability of research has been shown to be key to public opinion, financial support, and ethical execution of human subject research and use of animal models. Measures to ensure sustainability are now becoming clearly necessary for basic biology research from a biosecurity and educational perspective. Contrary to fears around the implementation of research guardrail institutions in the past, establishing guidelines for ethical research did not lead to hindrance of discoveries or, otherwise, prevent discoveries, at least in part due to self-regulation and unified leadership. Instead, it has guided researchers to help make these discoveries, with far less suffering to both human and animal subjects. Biosecurity education, crafted with the recently developed Tianjin Guidelines for Codes of Conduct for Scientists in mind, can help establish and perpetuate biosecurity norms among scientists that may minimize the chances of misuse of biology and biotechnology. Thus, merging the necessity of biosecurity education with research sustainability helps improve the contribution that ethically guided and secure science can have on society.

Together, there is ample opportunity to build biosecurity-centric competencies composed of knowledge, skills, and abilities into academic curricula for a sustainable future of biological research. Here, we propose 17 core biosecurity competencies organized into 6 themes. These competencies can be applied to all individuals, ranging from undergraduate and graduate students to postdoctoral researchers and principal investigators.

### Biosecurity history

Biological agents and toxins have been used in various conflicts throughout history with varying consequences. Biological weapon research and development started during the 20th century, where advances in microbiology and delivery systems contributed to notable, state-level biological weapon programs. Nonstate actors have also aspired to acquire and use pathogens to cause mass casualties. Recent progress in enabling technologies and decreasing costs associated with many research procedures and materials presents new hazards.

1. Understand and explain various biothreats and events throughout history.
2. Analyze the results and impacts of biothreat events.
3. Apply lessons learned to prevent, mitigate, and contain future biothreats.

### Bioethics

Certain laboratory experiments with pandemic pathogens and gene editing have permeated the public space in recent years, and while the value and risks of such work can be discussed, they do not necessarily breach ethical boundaries. However, we have witnessed horrifying and blatant bioethical transgressions in the past relating to the testing of deadly biological weapons on civilian populations. The future security-conscious scientists should also be ethically conscious.

4. Explain ethical transgressions in bioscience research.
5. Evaluate research proposals and ongoing projects through an ethical lens.

### Consequence-oriented thinking

While some experiments or studies may not cross ethical lines, the consequences of such work, including the public release of such work, could have detrimental effects—both in the tangible and intangible space. Can bad actors gain an advantage or ascertain critical methods, results, or inspiration from the results of a research study? Are there potential routes for accidental exposure or lapses in biosafety that could lead to the inadvertent release of a pathogen? What are the motivations for designing and conducting a research project, is it just for the science, and how might the surrounding community and public react when hearing about a specific study?

6. Assess research proposals for biosecurity risks arising from misuse or accidental releases.
7. Design experiments with a security-conscious approach.
8. Critically evaluate the motivation, necessity, and goals of experiments.
9. Assess public views on certain experiments.

### Leadership and communication

Researchers need to not only be scientifically proficient but also be effective leaders who communicate effectively. These qualities are important when addressing issues of biosecurity surrounding experiments and entire research studies. In the example of an academic research group, is the principal investigator initiating conversations about responsible science? Are they fostering an inclusive community among group members that will allow individuals to feel comfortable to speak up regarding experiments they perceive to be unnecessary or risky? Can the investigator approach issues with transparency, communicate well, and mediate potentially conflicting views regarding aspects of biosafety and biosecurity?

10. Display critical decision-making, team management, and decisive action when leading research groups, departments, and institutions.
11. Convey and embody messages of responsible, safe, and security-conscious scientific conduct.
12. Promote sustainable research practices.
13. Endorse scientific transparency during all stages of research.

### Policy in biotechnology and the sciences

In the modern era, science does not operate in a vacuum. Policies, regulations, norms, and perception all influence and govern the scientific space. Researchers should be aware of these policies and follow through, such as by reporting specific planned experiments to governing bodies or oversight committees before they are carried out. While students are typically part of organizations that are policy-aware, they should be able to recognize situations, both within and outside of their organization, when attempts to circumvent policies are made.

14. Understand the current rules and regulations surrounding biotechnology and the biosciences.
15. Promote rules-based approaches for biosecurity while maintaining and encouraging scientific freedom at various researches and governance.

### Humanity and scientific advancement

Many students enter the sciences with inherently noble intentions—curing diseases, saving lives through new approaches, and creating a safer and cleaner environment. However, they should also be aware of how certain scientific approaches, results, and associated advancements could become risks that threaten the health and well-being of society.

16. Recognize, prevent, or mitigate existential risks stemming from scientific advancements.
17. Promoting scientific discovery and probing of the natural order using safe and secure approaches.
